# Antibacterial properties of *Solenostemma argel* (Del.) Hayne against *Salmonella* strains from chicken meat: integrated GC–MS phytochemical profiling and molecular docking analysis

**DOI:** 10.3389/fnut.2025.1694017

**Published:** 2025-10-29

**Authors:** Abdulrahman Mohammed Alhudhaibi, Mahmoud Dahab, Hajo Idriss, Megren Faisal Almoteri, Emad M. Abdallah

**Affiliations:** ^1^Department of Biology, College of Science, Imam Mohammad Ibn Saud Islamic University (IMSIU), Riyadh, Saudi Arabia; ^2^Faculty of Pharmacy, Universiti Malaya, Kuala Lumpur, Malaysia; ^3^Deanship of Scientific Research, Imam Mohammad Ibn Saud Islamic University (IMSIU), Riyadh, Saudi Arabia; ^4^King Saud Hospital–Unayzah, Unayzah, Saudi Arabia; ^5^Department of Biology, College of Science, Qassim University, Buraydah, Saudi Arabia

**Keywords:** secondary metabolites, antibacterial activity, plants, natural products, computational biology, food-safety, poultry

## Abstract

**Background:**

*Salmonella enterica* is a leading cause of foodborne illness worldwide and poultry products, particularly chicken meat and there is growing interest in using plant extracts to protect chicken meat from Salmonella contamination during refrigeration. *Solenostemma argel* is a medicinal plant traditionally used to treat gastrointestinal disorders and quantitative data on its activity against *Salmonella enterica* are limited.

**Methods:**

Methanolic leaf extract was profiled by GC–MS to document major phytochemical classes. Antibacterial activity against clinical/retail *S. enterica* isolates was evaluated by disc diffusion and broth microdilution to determine inhibition zones, minimum inhibitory concentration (MIC) and minimum bactericidal concentration (MBC). A panel of GC–MS–identified constituents was subjected to *in silico* computational study based on oral-toxicity prediction and molecular docking against modelled outer-membrane proteins implicated in LPS assembly and membrane integrity (OmpV, LptE) to prioritize plausible membrane-active leads.

**Results:**

The methanolic extract displayed modest inhibition in disc-diffusion assays (mean inhibition zones were 7.8–9.7 mm). All *S. enterica* isolates shared a uniform MIC of 12.5 mg/ml; MBCs were strain-dependent (25 mg/ml for one isolate, 100 mg/ml for two isolates), yielding MBC/MIC ratios of 2–8 and indicating primarily bacteriostatic activity for some strains. GC–MS profiling revealed several volatile and semi-volatile metabolites of potential biological interest. However, it is important to note that some peaks, such as 1,3-dioxane and 2-[2-[2-(2-acetyloxyethoxy)ethoxy]ethoxy]ethanol, may represent laboratory-derived contaminants rather than genuine plant metabolites, emphasizing the need for rigorous contamination controls and complementary analytical approaches in future work. *In silico* docking prioritized a steroidal candidate [Estra-1,3,5(10)-trien-17β-ol] with the highest predicted affinities to OmpV (−7.9 kcal·mol^−1^) and LptE (−6.7 kcal·mol^−1^). Toxicity predictions suggested low oral toxicity for the majority of screened constituents.

**Conclusion:**

The methanolic leaf extract of *Solenostemma argel* exhibited moderate to weak *in vitro* anti-*Salmonella* activity, supported by computational docking results, and provide a foundation for further isolation, purification, and characterization of its active constituents.

## 1 Introduction

Food safety is a cornerstone of public health, playing a pivotal role in reducing morbidity and mortality, improving population well-being, and supporting economic development. Ensuring a safe food supply depends on rigorous scientific principles and effective regulatory frameworks. As technological advances reshape food production and distribution, updated regulations are essential to guarantee the availability of safe, nutritious foods that promote health across populations (1). Foodborne diseases remain a critical global health challenge, defined as illnesses arising from the consumption of food contaminated by pathogenic microorganisms, spoilage agents, toxins, or harmful chemicals. Contamination can occur at any stage of the food chain from primary production to final consumption (2). Major zoonotic bacterial pathogens responsible for significant illness and mortality include *Staphylococcus aureus*, *Salmonella* spp., *Campylobacter* spp., *Listeria monocytogenes*, and *Escherichia coli*, all of which are frequently linked to contaminated food products (3, 4).

Among these, *Salmonella* spp. is a leading cause of foodborne disease globally. In the European Union, Salmonella accounts for more than half of all reported foodborne outbreak cases. While poultry, livestock, and their products remain primary reservoirs, contamination is increasingly reported in diverse commodities, including dried foods, infant formula, fresh produce, and other ready-to-eat items (5). Non-typhoidal *Salmonella* (NTS) is a major public health burden, causing an estimated 93.8 million infections annually, with approximately 80.3 million linked to food consumption (6). Clinical manifestations range from self-limiting gastroenteritis, commonly caused by *Salmonella enterica* serovar Typhimurium, to more severe forms such as bacteremia, osteomyelitis, and reactive arthritis, often associated with *S. typhimurium* and *S. enteritidis* (*Salmonella enterica* subsp. *enterica* serovar Enteritidis). Enteric fever, caused by *S. typhi* and *S. paratyphi*, and asymptomatic carrier states further complicate control strategies (7, 8). The emergence and spread of antimicrobial resistance (AMR) in *Salmonella* strains is an escalating threat to effective treatment. Resistance is particularly alarming in isolates from agricultural sources, with high rates reported for sulfamethoxazole (91%), sulfonamides (51%), and ceftiofur (28.9%) (9).

Poultry and poultry products are recognized as major sources of *Salmonella*, a leading cause of serious foodborne infections in humans. Numerous poultry-associated *Salmonella* outbreaks have been documented worldwide, and the emergence of antimicrobial resistance in *Salmonella typhi* is an escalating concern, particularly across Asia and Africa (10). The contamination of food with *Salmonella* could be attributed to many reasons, such as improper hygiene during food processing, contaminated water, or poor agricultural practices. Interestingly, the reason varies between locations; non-typhoidal *Salmonella* contamination is widespread in poultry, meat, and vegetable products in India (11), while in Senegal, high contamination levels were reported in leafy greens such as mint, parsley, and lettuce, with supermarket samples showing higher prevalence than those from open markets (12). Unfortunately, the misuse of antibiotics in agriculture, including in livestock production and crop protection, has compounded the problem by facilitating the transfer of resistance genes along the food chain. Once ingested, these resistant bacteria can horizontally transfer resistance determinants to commensal or pathogenic bacteria in the human gut, further amplifying the antimicrobial-resistance crisis (13, 14). The computational (*in silico*) studies reported that, the outer membrane of *Salmonella* sp., specifically OmpV protein, are vital for its survival and interaction with hosts, contributes to membrane integrity and may be involved in nutrient acquisition and host-pathogen binding (15, 16). LptE is an important protein that helps build and transport lipopolysaccharide (LPS) to the outer membrane of bacteria (17, 18). Lack of LptE, the LPS cannot be properly inserted, then membrane can be weakened and make the bacteria more to stress and immune attacks. This makes LptE necessary for bacterial survival. Because both OmpV and LptE are indispensable for outer membrane function and virulence (19), they represent attractive molecular targets for the development of novel anti-*Salmonella* agents. Targeting such non-redundant, membrane-associated proteins offers a strategic approach to hinder bacterial viability and pathogenicity while reducing the likelihood of resistance development.

*Solenostemma argel* (Del.) Hayne is known as Hargal, an arid plant in the Apocynaceae family. It is the primary source of traditional medicine in a variety of countries, including Sudan, Egypt, Libya, Algeria and Saudi Arabia (20). In traditional medicine, almost all parts of *Solenostemma argel* (*S. argel*) are employed in traditional healing practices. Its leaves, bark, and stems are particularly valued for managing ailments such as diabetes, cardiovascular disorders, cough, gastrointestinal spasms, urinary tract infections, rheumatism, as well as liver and kidney diseases (21). Scientific investigations have validated several biological activities of *S. argel*, including antioxidant, anti-inflammatory, antimicrobial, anticancer, insecticidal, and nematocidal properties (22). *S. argel* is rich of some important phytochemicals, such as flavonoids, alkaloids, terpenoids, triterpenes, tannins and saponins (23). However, dose optimization in phytomedicine is essential, as high concentrations may cause hepatic and renal toxicity, whereas lower doses can retain therapeutic efficacy. In rats, doses of 600 mg/kg BW (BW, body weight) induced adverse liver and kidney effects, while lower doses remained beneficial (24). While some biological activities of *S. argel* have been documented, there is a clear paucity of recent studies on plants from Sudan, where distinct environmental factors may alter their phytochemical composition and associated bioactivities. Therefore, the present study aimed to evaluate the antibacterial potential of *S. argel* against *Salmonella* strains isolated from chicken meat, while characterizing its phytochemical composition using GC–MS analysis. Furthermore, the study investigated the interactions of key bioactive compounds with essential *Salmonella* outer membrane proteins, OmpV (Outer membrane protein V) and LptE (Lipopolysaccharide transport protein E), through molecular docking, integrating experimental and computational approaches to elucidate the mechanistic basis of its anti-*Salmonella* activity and assess its potential as a natural agent for controlling *Salmonella* contamination in poultry products.

## 2 Materials and methods

### 2.1 Plant collection and extraction

Leaves of *S. argel* cultivated in Gezira, central Sudan, were collected in 2023 and scientifically identified by a qualified botanist. The plant material was air-dried and stored in well-sealed plastic bags until further use. Prior to extraction, the dried leaves were ground into a fine powder using a mechanical blender. A 50 g portion of the powdered material was macerated in 500 ml of 80% methanol for 7 days with intermittent shaking. The mixture was filtered twice through Whatman No. 1 filter paper, and the filtrate was incubated at 40°C for approximately 2 weeks to allow complete solvent evaporation. The resulting dried methanolic extract was carefully scraped, collected under aseptic conditions, and stored in a clean, airtight container in the fridge until reconstitution for experimental use (25). The dried extract was reconstituted in 80% methanol for antibacterial testing, which does not affect bacterial growth.

### 2.2 GC–MS analysis

The gas chromatography–mass spectrometry (GC–MS) investigation of the methanolic leaf extract of *S. argel* was conducted using a Perkin-Elmer Clarus 680 system (Perkin-Elmer, Inc., USA) coupled with an Elite-5MS fused silica capillary column (30 m length × 250 μm internal diameter × 0.25 μm film thickness). Helium gas of high purity (99.99%) was used as the carrier at a constant flow rate of 1.0 ml/min. The injection volume was 1 μl, applied in split mode at a 10:1 ratio, with the injector temperature set at 250°C. The column oven temperature was originally set at 50°C for 3 min, increased at a rate of 10°C/min to 280°C, and then elevated to 300°C, which was sustained for 10 min. Electron ionization was performed at 70 eV with a scan duration of 0.2 s, capturing mass fragments within the range of 40–600 m/z. The identification of phytochemical ingredients was achieved by comparing retention times, peak areas, peak heights, and mass spectral fragmentation patterns with reference compounds in the National Institute of Standards and Technology (NIST) spectrum library (26).

### 2.3 Bacterial strain isolation and characterization

Chilled chicken samples were purchased from multiple retail outlets in Riyadh, Saudi Arabia, and transported to the laboratory in insulated coolers to maintain the cold chain. On receipt, the external surface of each primary package was disinfected with an appropriate surface sterilant, and the package was aseptically opened. Approximately 0.1 ml of the exudate fluid present within each package was aseptically aspirated using a sterile 1 ml syringe and immediately inoculated onto selective/differential media, including Salmonella–Shigella (SS) agar and xylose lysine deoxycholate (XLD) agar, to facilitate the isolation of *Salmonella* spp.

A sterile loop was used to streak a single drop of sample onto each plate using a four-quadrant streaking technique to obtain well-separated colonies. Inoculated plates were incubated inverted at 37°C for 24 h. Presumptive *Salmonella* colonies were sub-cultured onto nonselective agar to obtain pure cultures. Gram staining was performed on pure isolates as part of routine preliminary characterization.

### 2.4 Automated identification and antibiotics profiling

Presumptive *Salmonella* isolates obtained through routine cultural and biochemical procedures were subjected to confirmatory testing using the MicroScan WalkAway 96 Plus system (Beckman Coulter, USA). Pure colonies from 24-h cultures grown on blood agar were suspended in sterile saline to match a 0.5 McFarland turbidity standard and subsequently inoculated into the Negative Combo Panel Type 44 (PN B1017-207). The system, which integrates conventional biochemical reactions with fluorogenic substrates, was operated at 35 ± 2°C for 18–24 h. Isolates were identified at the species level, and confirmation as *S. enterica* was accepted when identification probability was ≥90%. For antibacterial susceptibility testing, the same standardized inoculum was dispensed into the Negative MIC Panel Type 44 (PN B1017-207) according to the manufacturer’s instructions and following CLSI recommendations. Panels were incubated within the WalkAway 96 Plus system at 35 ± 2°C for 16–20 h. Minimum inhibitory concentrations (MICs) were automatically calculated by the instrument and interpreted according to CLSI breakpoints (27). The antibiotics tested included 25 to 27 antibiotics (Supplementary Figure 1). Prior to experimental procedures, quality control was routinely verified using *E. coli* ATCC 25922 and *P. aeruginosa* ATCC 27853 reference strains.

### 2.5 Disc diffusion assay

The antibacterial activity of the methanolic extract of *S. argel* against the 3 isolated and identified *Salmonella* strains. Collected from chicken was evaluated using the disc diffusion method, adapted with minor modifications from a previously described methodology (28), with minor modifications. Briefly, bacterial suspensions were adjusted to approximately 1 × 10^6^ CFU/ml and uniformly spread over the surface of Mueller-Hinton agar plates using sterile cotton swabs. Sterile filter paper discs (6 mm diameter) saturated with 10 μl of the methanol extract (at 200 mg/ml) were placed onto the inoculated plates, under aseptic conditions. Blank discs saturated with chloramphenicol (2.5 mg/ml) were loaded as positive controls. Prior to the experiment, 80% methanol was tested and showed no inhibitory effect on bacterial growth. The plates were incubated at 37°C for 24 h. Following incubation, inhibition zone diameters were measured in millimeters. All assays were performed in triplicate, and the results were expressed as mean values ± standard deviation.

### 2.6 Determination of MIC and MBC values

The minimum inhibitory concentration (MIC) of the methanol extract of *S. argel* against the tested bacteria was determined using the microbroth dilution method in sterile 96-well microplates, as previously described in standardized protocols. Briefly, each well was filled with 95 μl of double-strength Mueller-Hinton broth, followed by serial dilutions of the test sample (dissolved in 5% DMSO) and chloramphenicol as the reference antibiotic. Subsequently, 10 μl of bacterial suspensions adjusted to 10^6^ CFU/ml were inoculated into each well. The plates were incubated at 37°C for 24 h. Following incubation, 40 μl of a TTC solution (0.2 μg/ml) was added, and the plates were re-incubated for 2 h. A color change from colorless to red indicated microbial activity. The MIC was defined as the lowest concentration at which no visible bacterial growth was observed. The minimum bactericidal concentration (MBC) was determined by sub-culturing 50 μl aliquots from wells corresponding to the MIC and higher concentrations onto Mueller-Hinton agar plates. The plates were incubated at 37°C for 24 h. The MBC was defined as the lowest concentration that completely inhibited bacterial growth. The MBC/MIC ratio was subsequently calculated to distinguish between bacteriostatic and bactericidal activity (29).

### 2.7 Computational studies of S. argel compounds

#### 2.7.1 Toxicity assessment of *S. argel*-derived compounds

To assess the potential toxicity of compounds isolated from *S. argel*, the ProTox 3.0 online platform^1^ was utilized (accessed on 7 July 2025), as described by (30). This tool applies integrated computational models to predict a variety of toxicological endpoints. The chemical structures of the selected compounds were first converted into simplified molecular input line entry system (SMILES) format before submission (Supplementary Table S1).

#### 2.7.2 Protein target acquisition and structure modeling

Since crystal structures for the target outer membrane proteins, namely OmpV from *S. typhimurium* (MipA/OmpV family protein, UniProt ID: A0A634SAW8) and LptE from *S. enteritidis* (LPS-assembly lipoprotein LptE, UniProt ID: A0A5W2VQP4), were unavailable in the protein data bank (PDB), their respective amino acid sequences were retrieved from UniProt^2^ in FASTA format. The three-dimensional structures of these proteins were subsequently generated through homology modeling using the Swiss-Model web tools^3^ (accessed on 7 July 2025).

#### 2.7.3 Refinement and validation of *Salmonella* outer membrane protein models

To enhance the structural accuracy of the predicted outer membrane proteins, refinement was performed using the ModRefiner tool. The quality of the refined models was evaluated through Ramachandran plot analysis and further validated using the ERRAT and PROCHECK programs, accessed via the SAVES v6.1 server^4^ (accessed on 13 July 2025).

#### 2.7.4 Binding pocket identification and drug ability assessment

Potential ligand-binding sites within the selected protein structures were identified and analyzed using FPocketWeb 1.0.1 (accessed on 13 July 2025), a web-based tool that applies the FPocket algorithm to detect and evaluate druggable pockets in protein models.

#### 2.7.5 Molecular interaction analysis

Molecular docking of ligands with target receptors was conducted using the CB-Dock2 web server^5^ (31) (accessed on 10 July 2025), which integrates cavity detection and template-based docking to enhance prediction reliability. To ensure reproducibility, the center coordinates and size of the primary docking cavity identified by CB-Dock2 for each protein are provided in Supplementary Table S8. The protonation states of ionizable residues and ligands were assigned by default for a physiological pH of 7.4 prior to docking simulations. Blind docking was performed, and the conformations with the highest binding affinities were selected for further interaction analysis with the proteins’ amino acid residues. Visualization of the docking results was carried out using Discovery Studio Visualizer (v21.1.0.20298) for 2D interaction diagrams and UCSF ChimeraX (v1.8) for 3D structural representations.

### 2.8 Statistical analysis

The results were presented as the mean ± standard deviation (SD) and all experiments were conducted in triplicate (n = 3). GraphPad Prism 9 and XLSTAT v.2016 were employed to conduct statistical analyses. One-way analysis of variance (ANOVA) was followed by Tukey’s *post hoc* test to evaluate mean differences, with *p* < 0.05 being considered statistically significant.

## 3 Results and discussion

### 3.1 Characterization of bioactive compounds

**Figure 1:**
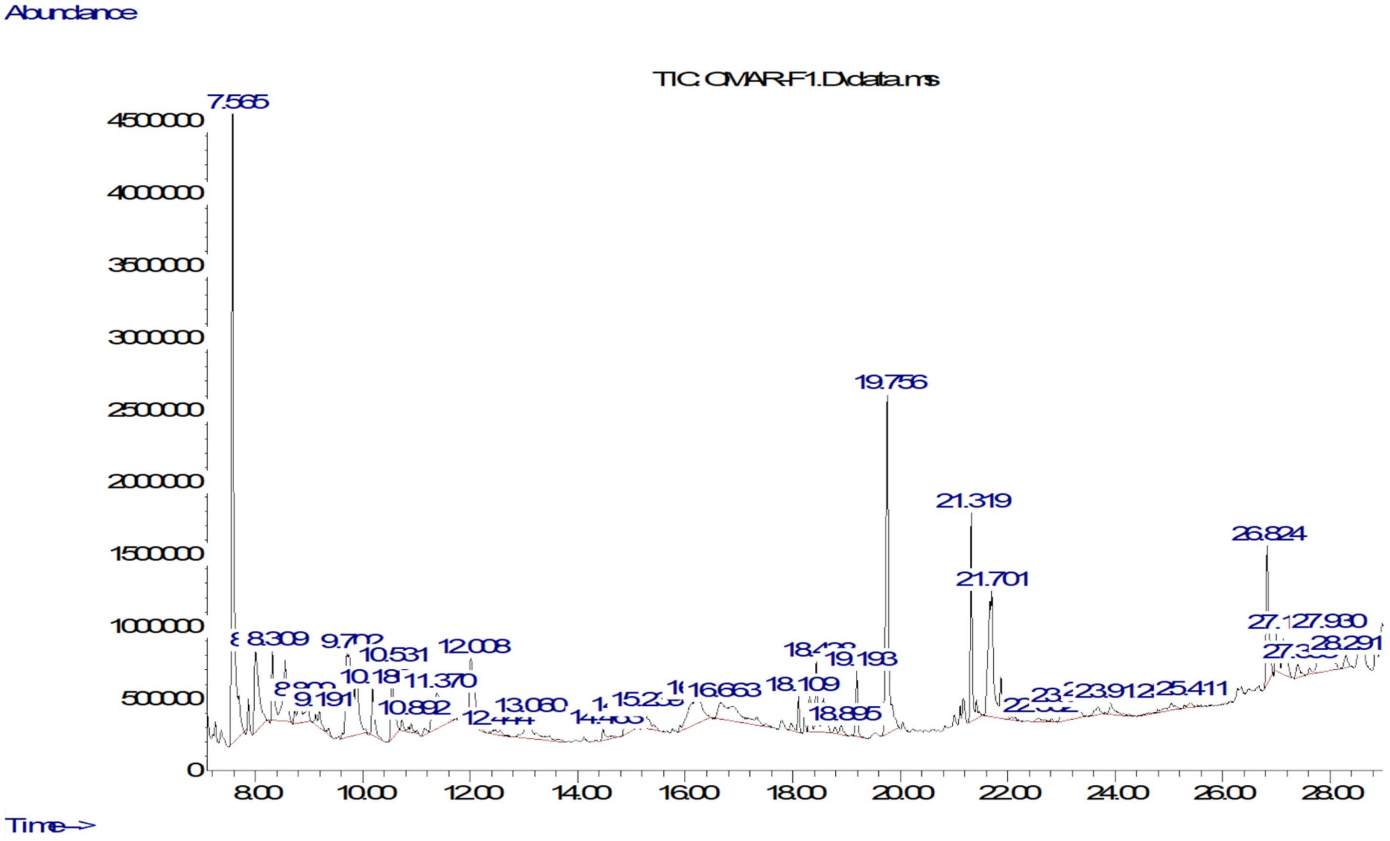
GC–MS chromatogram of the methanol extract of *S. argel*.

The GC–MS analysis of the extract of *S. argel* methanolic extract demonstrated a chemical diversity, involving the identification of 32 compounds achieved by employing multiple phytochemical classes including phenolic compounds, fatty acids and derivatives, esters and alcohols, terpenes and steroids, and multiple classes of “others” (Table 1). The compounds were identified by mass spectral matching with the NIST library and ranking based on their normalized peak area (%) indication representing relative abundance (32). The Chromatogram in Figure 1 was in reasonable agreement with the tabled data, particularly for the highest abundance species. Although 1,3-dioxane was clearly detected in the chromatogram with a relatively high peak area (14.07%), it should not be considered a natural component of the plant. These types of chemicals typically originate from external sources, such as leftover solvents or inexpensive plastic containers used during sample collection, storage, or preparation. Therefore, these signals were treated as possible contaminants (not from the plant) instead of natural plant components. The implications of these potential contaminants for phytochemical profiling are discussed in the section on study limitations. Equally, the peak at RT = 19.755 min identified Hexadecanoic acid, methyl ester, a saturated fatty acid from plants known to exhibit antibacterial properties, was accurate with both the peak and displayed in the data. Interestingly, separate peaks in the RT = 25.8 to 27.4 min range were identified as long-chain unsaturated fatty acids including Oleic acid, 2-Methyl-Z,Z,Z-3,13-octadecadienol (33). These compounds are known for their ability to disrupt microbial and fungal membranes, thus enabling the biological activity of the extract. Phytochemically, the analysis of accumulated concentrations indicates that phenolic compounds are the strongest group at 34.31% of the entire composition as provided in Table 2 (34). The group contains phenolic compounds that feature bioactivity data, such as Phenol, 2,4-bis(1,1-dimethylethyl)-, 2-Methoxy-4-vinylphenol, and (E)-Stilbene. The second strongest class is “Others” at 22.59%, which also includes various cyclic compounds and possibly synthetic compounds that may be artifacts or misidentified. The next component was fatty acids, and fatty acids and phosphates, at 19.26% of the total. Fatty acids include some highly biologically active molecules or fatty acids: Hexadecanoic acid, Oleic acid, and Linolenic acid (35). The next two components were esters and alcohols at 9.14%, and terpenes and steroids at 8.90%. This group includes compounds such as Phytol and Estra-1,3,5(10)-trien-17β-ol. Overall, the classes of compounds support the traditional uses of the plant, considering that known and bioactive compounds are generally understood to permeate the microbial membranes to interrupt membrane integrity and inactivate oxidative enzymes (36). Fatty acids of the plant provide the ability to destabilize the bacterial cell envelope. Both Phytol and Oleic acid are reported as antifungal and antibacterial compounds (37). Overall these types of chemical processes and compounds provide plausible support for ethnobotanically based use of *Rhazya stricta* in the treatment of infections and skin conditions (38). However, there are four compounds that may be problematic in respect to their authenticity. These include: 2-[2-[2-[2-(2-Acetyloxyethoxy)ethoxy]ethoxy]ethanol and Trimethyl 1,3,3-oxydipropanoate which are not commonly reported in plants and likely included as residual solvents. Potentially some of these compounds may stem from plastic substrates or PCR tubes that are disposable products used in contemporary laboratory techniques. However, there are additional sugars such as D-Galactose, 6-deoxy- and 1-alpha-D-Glucopyranoside, methyl that normally require derivatization prior to GC–MS detection and therefore may also be incorrectly classified due to the usage of a library of products (39). Overall, the GC–MS results show a good representation of the plant’s potential chemical activity as they relate to phenolic compounds and fatty acid phosphates.

**Table 1:** Chemical compounds identified in the methanolic extract of *S. argel* using GC–MS analysis.

Compound	Phytochemical class	Mol weight	RT (min)	Area	Absolute height	Area (%)
1,3-Dioxane	Others	88.052	7.565	13,444,078	4,707,884	14.07
Phenol, 2,4-bis(1,1-dimethylethyl)-	Phenolic Compounds	206.32	8.001	11,424,914	378,634	11.96
Estra-1,3,5(10)-trien-17.beta.-ol	Terpenes and Steroids	270.37	8.309	8,053,841	1,232,894	8.43
4H-Pyran-4-one, 2,3-dihydro-3,5-dihydroxy-	Phenolic Compounds	128.1	8.809	7,352,661	815,127	7.7
Hexadecanoic acid, methyl ester	Fatty Acids and Derivatives	270.45	9.191	6,984,525	1,245,599	7.31
Phytol	Esters and Alcohols	296.53	9.702	3,841,973	440,999	4.02
Oleic Acid	Fatty Acids and Derivatives	282.47	10.18	3,698,228	550,326	3.87
2-Methoxy-4-vinylphenol	Phenolic Compounds	150.17	10.531	3,658,127	584,274	3.83
2-Furanmethanol, 5-ethyltetrahydro-	Phenolic Compounds	116.073	10.892	3,575,890	830,048	3.74
2-Furancarboxaldehyde, 5-(hydroxymethyl)-	Phenolic Compounds	126.11	11.37	3,481,926	539,467	3.64
2-[2-[2-[2-(2-Acetyloxyethoxy)ethoxy]ethoxy]ethanol	Others	410.225	12.008	3,370,947	828,786	3.53
2-Acetylamino-3-hydroxy-propionic acid	Alkaloids	119.11	12.444	3,318,852	718,600	3.47
(E)-Stilbene	Phenolic Compounds	180.25	13.06	3,291,431	473,110	3.44
12-Octadecadienoyl chloride, (Z, Z)-	Fatty Acids and Derivatives	298.16	14.463	3,069,228	486,392	3.21
Oxeprime, 2,7-dimethyl-	Others	272.44	14.878	2,118,339	920,014	2.22
2-Propanol, 1-(1-methylethoxy)-	Esters and Alcohols	104.15	15.239	2,056,957	717,840	2.15
2-Methyl-Z, Z, Z-3,13-octadecadienol	Fatty Acids and Derivatives	280.5	16.27	1,774,904	420,927	1.86
n-Hexadecanoic acid	Fatty Acids and Derivatives	256.42	16.663	1,544,173	479,010	1.62
9,12,15-Octadecatrienoic acid, (Z, Z, Z)-	Fatty Acids and Derivatives	278.27	18.109	1,324,866	507,245	1.39
Trimethyl 1,3,3-oxydipropanoate	Esters and Alcohols	190.19	18.438	1,130,752	850,422	1.18
Pentanoic acid, 3-methyl-, methyl ester	Esters and Alcohols	116.16	18.895	1,101,396	577,088	1.15
D-Galactose, 6-deoxy-	Carbohydrates and Derivatives	164.16	19.193	955,455	413,665	1
1-alpha-D-Glucopyranoside, methyl	Carbohydrates and Derivatives	194.18	19.756	732,924	554,502	0.77
2,3-Dimethyldecane	Others	170.33	21.319	732,267	473,696	0.77
11-Dodecen-1-ol acetate	Esters and Alcohols	212.33	21.701	615,769	466,999	0.64
Acetamide, N-(3-oxo-4-isoxazolidinyl), (R)-	Alkaloids	144.12	23.083	526,131	337,788	0.55
Tricyclo[7.1.0.0(4,6)]decane-5-carboxamide	Others	207.16	23.678	487,079	743,358	0.51
Cyclopropane carboxamide, 2-cyclopropyl-2-methyl-	Others	207.16	23.912	487,079	743,358	0.51
Bicyclo[3.1.1]heptane, 2,6,6-trimethyl-	Terpenes and Steroids	138.2	25.049	449,445	314,250	0.47
1,3-Cyclohexanediol, 5-methyl-2-(1-methylethyl)-	Others	158.25	25.411	418,936	286,447	0.44
6-Methyl-2,3-dihydro-pyran-2,4-dione	Others	128.1	26.824	364,922	284,971	0.38
Oxirane, 2-methyl-2-(1-methylethyl)-	Others	100.16	27.122	155,139	482,516	0.16

**Table 2:** Percentage composition of phytochemical classes identified in the methanolic extract of *S. argel* by GC–MS analysis.

Phytochemical class	The percentage (%)
Phenolic Compounds	34.31
Others	22.59
Fatty Acids and Derivatives	19.26
Esters and Alcohols	9.14
Terpenes and Steroids	8.9
Alkaloids	4.02
Carbohydrates and Derivatives	1.77

### 3.2 Bacteriological assessment

Automated identification and antibiotic susceptibility profiling of the presumptive *Salmonella* isolates yielded notable findings (Supplementary Figure S1). Among the four tested samples, sample 1 was identified as *Proteus mirabilis* rather than *Salmonella* spp., while the remaining three were confirmed as *Salmonella enterica*. The antibiotic profiling demonstrated that *P. mirabilis* was susceptible to all 25 tested antibiotics, suggesting that this isolate likely represents a commensal member of the chicken intestinal microbiota. Its presence on poultry meat is therefore most plausibly attributed to fecal contamination during slaughtering processes. Although *P. mirabilis* is not recognized as a primary poultry pathogen, it may contribute to cellulitis in broilers and, importantly, has been implicated in human infections following the consumption of contaminated or undercooked poultry (40). These results highlight its potential role as a food safety concern, emphasizing the need for stringent hygiene measures during poultry processing to mitigate risks of cross-contamination and subsequent transmission to consumers. The other samples (number 2, 3, and 4) were all confirmed to be *Salmonella enterica*. Antibiotic susceptibility profiling revealed that among 25 antibiotics tested, sample 2 was resistant to 6 antibiotics, sample 3 was resistant to 2 antibiotics, while sample 4 was resistant to 6 different antibiotics. These results indicate that samples 2 and 4 are multi-drug resistant (MDR) *Salmonella enterica* strains. MDR bacteria are widely defined as acquired non-susceptibility to at least one antimicrobial agent in three or more distinct antimicrobial classes (41).

### 3.3 Anti-*Salmonella* activity of *S. argel*

The results of the disc-diffusion test are shown in Table 3 and (Supplementary Figure S2). The antibacterial activity of *S. argel* methanol extract compared to chloramphenicol (as a positive control) was assessed against *Proteus mirabilis* and *Salmonella enterica* using the disc diffusion method. The extract exhibited inhibition zones ranging from 7.83 ± 0.29 to 10.0 ± 1.0 mm. Statistical analysis revealed that the extract produced comparable inhibitory effects against *P. mirabilis* and two *S. enterica* isolates (Samples 1–3; *p* > 0.05), whereas the inhibition observed against the fourth *S. enterica* isolate was significantly lower (*p* < 0.05).

**Table 3:** Antibacterial activity of *S. argel* methanol extract against the selected strains using disc diffusion method.^*^

Sample number	Bacterial strain	Mean inhibition zones (mm)	
		*Solenostemma argel* methanol extract (10 μl/disc from 100 mg/ml)	Chloramphemicol (10 μl/disc from 5 mg/ml)
1	*Proteus mirabilis*	10.0 ± 1.0ᵃ	33.33 ± 0.58ᵃ
2	*Salmonella enterica*	9.67 ± 0.58ᵃ	7.50 ± 0.50ᶜ
3	*Salmonella enterica*	9.33 ± 0.58ᵃ	23.33 ± 0.58ᵇ
4	*Salmonella enterica*	7.83 ± 0.29ᵇ	8.33 ± 0.58ᶜ

Data are presented as the mean ± standard deviation of three replicates (*n* = 3). Mean values labeled with dissimilar letters are significantly different according to Tukey’s honestly significant difference test following one-way ANOVA (*p* < 0.05).

* The final bacterial density reached approximately 10^6^ CFU/ml.

In contrast, chloramphenicol demonstrated markedly higher inhibition against *P. mirabilis* (33.33 ± 0.58 mm) compared with all *S. enterica* isolates (*p* < 0.05). Among *S. enterica*, the response was strain-dependent: one isolate exhibited intermediate sensitivity (23.33 ± 0.58 mm), while the remaining two were significantly less susceptible (7.50 ± 0.50 and 8.33 ± 0.58 mm). These findings highlight the consistent but moderate antibacterial effect of *S. argel* extract across strains, while chloramphenicol showed variable activity ranging from strong inhibition of *P. mirabilis* to markedly reduced efficacy against certain *S. enterica* isolates. To our knowledge, this study presents the first investigation into the anti-*Salmonella* activity of *S. argel* specifically against *Salmonella enterica*. Existing reports indicate weak to negligible activity, the aqueous, butanol, and ethyl acetate extracts of *S. argel* were reported to exhibit no antibacterial activity. In contrast, the diethyl ether extract demonstrated moderate efficacy against *P. aeruginosa* ATCC 27853 (42). The aqueous extract of *S. argel* exhibited a 17 mm inhibition zone against *Escherichia coli* at a concentration of 25% (w/v) (43). The ethanolic leaf extract of *S. argel* exhibited superior antibacterial activity against *Klebsiella pneumoniae* compared to its methanolic counterpart (44). Consequently, methanol may not be the optimal solvent for the efficient extraction of much antibacterial compounds from this plant.

As shown in Table 4 and Supplementary Figure S3, the MIC of *S. argel* methanolic extract was consistent across all tested isolates at 12.5 mg/ml. However, the MBC varied between 25 and 100 mg/ml. Consequently, the calculated MBC/MIC ratios ranged from 2 to 8. Ratios of 2 observed against *P. mirabilis* and one *S. enterica* isolate indicated bactericidal activity, while ratios of 8 in two *S. enterica* isolates suggested primarily bacteriostatic effects. These findings highlight that the extract exerted growth inhibition at a uniform MIC, but bactericidal activity was strain-dependent and required higher concentrations in certain isolates. When compared with the positive control chloramphenicol, the extract displayed a weaker antibacterial activity profile. Chloramphenicol exhibited much lower MIC values (<0.078–0.312 mg/ml) but showed variable bactericidal capacity: one isolate demonstrated a clear bactericidal effect (MBC/MIC ≥1), whereas others were bacteriostatic with ratios exceeding 4, including one as high as ~20. This comparison demonstrates that, while chloramphenicol exhibited markedly greater potency, the *S. argel* extract was still capable of exerting bactericidal activity against selected strains, although at comparatively higher concentrations. These findings highlight its possibility of being supplementary antimicrobial source and emphasize the need for purification and characterization of its active constituents in future investigations, which may reveal compounds with enhanced and more consistent antibacterial efficacy. Our assumption is based on the fact that a tested compound is classified as bactericidal if the MBC/MIC ratio is ≤4.0. Conversely, a ratio exceeding 4.0 indicates bacteriostatic/fungistatic properties (45). Importantly, the disparity between the extract and chloramphenicol highlights the limited potency of *S. argel* compared with conventional antibiotics, although its activity against multidrug-resistant pathogens remains noteworthy. Previous research on *S. argel*’s antibacterial properties has largely focused on broader bacterial targets or used different extraction methods. The reported MIC of 12.5 mg/ml for *S. argel* is notably high, indicating weak antimicrobial activity. In natural product research, plant extracts with MIC values above 1.5 mg/ml are generally considered to have poor or negligible activity. For instance, extracts with MIC values between 0.6 and 1.5 mg/ml are classified as moderate inhibitors, while those above 1.5 mg/ml are regarded as weak inhibitors (46).

**Table 4:** The results of MIC and MBC tests of *S. argel* methanol extract compared to the positive control (chloramphenicol).

Sample number	Bacterial strain	*Solenostemma argel* methanol extract (mg/ml)			Effect of extract	Chloramphenicol (mg/ml)			Effect of antibiotic
		MIC	MBC	MBC/MIC		MIC	MBC	MBC/MIC	
1	*Proteus mirabilis*	12.5	25	2	Bactericidal	<0.078	1.25	>16.0	Bacteriostatic
2	*Salmonella enterica*	12.5	25	2	Bactericidal	0.312	1.25	4.01	Bacteriostatic
3	*Salmonella enterica*	12.5	100	8	Bacteriostatic	<0.078	0.078	≥1.0	Bactericidal
4	*Salmonella enterica*	12.5	100	8	Bacteriostatic	0.312	6.25	20.03	Bacteriostatic

Sulieman et al. (47) examined aqueous extracts of *S. argel* against Gram-negative bacteria, including *Salmonella typhi*, reporting successful inhibition in disc diffusion assays. However, MIC/MBC data were not provided, limiting quantitative comparison in terms of potency (47). A comprehensive review of *S. argel*’s phytochemical and pharmacological properties described its general antimicrobial potential, but with minimal emphasis on quantitative anti-*Salmonella* data (20). Future investigations should focus on isolating the antibacterial constituents of *S. argel* using diverse solvents and extraction methodologies. In addition, *in vivo* studies are essential to validate and substantiate the traditional use of this plant in treating abdominal disorders.

On the other side, while our results provide useful baseline data on the antibacterial properties of *S. argel* extract, it is crucial to situate them within the broader challenge of multidrug-resistant (MDR) bacterial strains and to explore possible mechanistic implications. MDR in *Salmonella* and other gram-negative pathogens is increasingly common, reducing therapeutic options and increasing urgency for novel antimicrobials such as ESBL-producing *Salmonella* (48). The variation we observed in extract sensitivity among different *S. enterica* isolates, some being bactericidal at moderate doses, others only bacteriostatic at much higher concentrations, likely reflects genetic or phenotypic heterogeneity, possibly including differences in outer membrane structure, efflux pump activity, or other resistance determinants. Moreover, our GC–MS profiling suggests the presence of both phenolic acids (or related phenolic compounds) and fatty acids among the volatile or semi-volatile constituents. Phenolic compounds, such as gallic acid, protocatechuic acid, and vanillic acid, have been shown in *S. typhimurium* to disrupt the bacterial outer membrane, lead to membrane permeabilization, inhibit virulence gene expression, and affect growth even in MDR pathogens (49). Free fatty acids are likewise reported in the literature to act via multiple mechanisms: destabilizing membranes, uncoupling oxidative phosphorylation, interrupting electron transport chains, and inhibiting nutrient uptake (50). Accordingly, the antibacterial activity of *S. argel* extract likely arises from both independent and synergistic actions of phenolics and fatty acids: phenolics may disrupt membrane integrity or regulatory elements (e.g., virulence factors, outer membrane proteins), while fatty acids could enhance this effect by further permeabilizing membranes or inducing metabolic dysfunction. This synergy may explain why some strains are killed at lower concentrations, whereas others are only inhibited at higher doses. Future work should fractionate the extract to test phenolic and fatty acid fractions individually and in combination, alongside profiling bacterial resistance determinants. Such studies would clarify strain-specific responses and could guide optimized formulations to overcome MDR phenotypes.

### 3.4 Computational assessment

A non-toxic rating for oral use is crucial for drug candidates, particularly those taken orally, as it reduces the risk of adverse in the body. *In silico* toxicity analysis of 19 *S. argel*-derived compounds was showed a mainly favorable safety profile (Table 5), with the majority falling into toxicity classes 4 to 6, based on LD₅₀ values predicted by ProTox-II. (E)-Stilbene, Hexadecanoic acid methyl ester, and 2-[2-[2-[2-(2-Acetyloxyethoxy)ethoxy]ethoxy]ethanol compounds were classified as non-toxic (Class 6) with 100% prediction accuracy. Organ toxicity predictions were showed most compounds inactive for hepatotoxicity, immunotoxicity, mutagenicity, and cytotoxicity (Supplementary Table S2). While a few compounds such as Oleic acid (Class 2) and 2-Furancarboxaldehyde, 5-(hydroxymethyl)- (Class 3) showed higher oral toxicity risks, their organ toxicity scores remained largely inactive. Compound 3 (Estra-1,3,5(10)-trien-17β-ol) demonstrated no detectable toxicity (LD50 = 5,010 mg/kg, Class 6) with strong binding ability, this compound shows great promise as a drug candidate. To confirm the predicted low-toxicity profile of the identified compounds, further studies should therefore include experimental assays to validate cytotoxicity, organ-specific toxicity, and systemic safety, before considering clinical translation.

**Table 5:** Predicted oral toxicity of *S. argel* compounds from ProTox-II web server.

Compound name	LD50 (mg/kg)	Predicted toxicity class^*^	Average similarity (%)	Prediction accuracy (%)
1,3-Dioxane	2,000	4	71.9	69.26
Phenol, 2,4-bis(1,1-dimethylethyl)-	2,820	5	188.31	70.97
Estra-1,3,5(10)-trien-17β-ol	5,010	6	77.18	68.26
4H-Pyran-4-one, 2,3-dihydro-3,5-dihydroxy-	595	4	60.38	68.07
Hexadecanoic acid, methyl ester	5,000	5	100	100
Phytol	1,016	4	76.9	69.26
Oleic Acid	48	2	100	100
2-Methoxy-4-vinylphenol	1,920	4	85.3	70.97
2-Furancarboxaldehyde, 5-(hydroxymethyl)-	79	3	47.53	54.26
2-[2-[2-[2-(2-Acetyloxyethoxy)ethoxy]ethoxy]ethanol	11,000	6	100	100
2-Acetylamino-3-hydroxy-propionic acid	1,300	4	61.83	68.07
(E)-Stilbene	920	4	100	100
12-Octadecadienoyl chloride, (Z, Z)-	5,000	5	70.79	69.26
2-Propanol, 1-(1-methylethoxy)-	4,400	5	100	100
2-Methyl-Z, Z, Z-3,13-octadecadienol	1,016	4	81.36	70.97
n-Hexadecanoic acid	900	4	100	100
9,12,15-Octadecatrienoic acid	4,260	5	79.3	69.26
Pentanoic acid, 3-methyl-, methyl ester	5,000	5	87.5	70.97
6-Methyl-2,3-dihydro-pyran-2,4-dione	595	4	60.38	68.07

* Class 1: fatal if swallowed (LD50 ≤ 5); Class 2: fatal if swallowed (5 < LD50 ≤ 50); Class 3: toxic if swallowed (50 < LD50 ≤ 300); Class 4: harmful if swallowed (300 < LD50 ≤ 2,000); Class 5: may be harmful if swallowed (2,000 < LD50 ≤ 5,000); Class 6: nontoxic (LD50 > 5,000).

The *S. argel* compounds displayed varied physicochemical properties (Table 6), with LogP values ranging from 0.46 to 4.99. Ten compounds were showed favorable oral bioavailability. Most compounds complied with RO5, suggesting potential for oral bioavailability. LogP values ranged from 0.46 to 4.99, with acceptable hydrogen bond donor and acceptor counts across most compounds, highlight its relevance for future pharmacological and bioavailability studies. Notably, Estra-1,3,5(10)-trien-17β-ol, a promising docking hit, showed only one RO5 violation, possessing a LogP of 2.94 and 1 hydrogen bond donor and acceptor.

**Table 6:** Physicochemical properties and drug-likeness of *S. argel* compounds.

Compound name	Hydrogen bonds		LogP^*^ (iLogPo/w)	Molar refractivity	RO5 violation^**^
	Acceptor Donor				
1,3-Dioxane	2	0	1.49	21.4	0
Phenol, 2,4-bis(1,1-dimethylethyl)-	1	1	2.65	67	0
Estra-1,3,5(10)-trien-17β-ol	1	1	2.94	79	1
4H-Pyran-4-one, 2,3-dihydro-3,5-dihydroxy-	4	2	1.19	32.39	0
Hexadecanoic acid, methyl ester	2	0	4.41	85.12	1
Phytol	1	1	4.48	97.99	1
Oleic Acid	2	1	3.8	85.13	1
2-Methoxy-4-vinylphenol	1	0	2.29	43.02	0
2-Furancarboxaldehyde, 5-(hydroxymethyl)-	3	1	0.94	30.22	0
2-[2-[2-[2-(2-Acetyloxyethoxy)ethoxy] ethoxy]ethanol	5	1	2.37	45.19	0
2-Acetylamino-3-hydroxy-propionic acid	4	3	0.46	32.08	0
(E)-Stilbene	0	0	2.72	61.81	1
12-Octadecadienoyl chloride, (Z,Z)-	1	0	4.5	92.69	1
2-Propanol, 1-(1-methylethoxy)-	2	1	2.12	33.2	0
2-Methyl-Z,Z,Z-3,13-octadecadienol	1	1	4.99	93.66	1
n-Hexadecanoic acid	2	1	3.85	80.8	1
9,12,15-Octadecatrienoic acid	2	1	3.62	79.38	0
Pentanoic acid, 3-methyl-, methyl ester	2	0	2.3	37.05	0
6-Methyl-2,3-dihydro-pyran-2,4-dione	4	2	1.19	32.39	0

* Octanol–water partition coefficient.

** Lipinski rule of five.

The structural characteristics of the Salmonella outer membrane proteins, OmpV and LptE (LPS), were predicted using AlphaFold2, with details summarized in Supplementary Table S3. OmpV and LptE proteins were modeled as monomers using the AlphaFold v2 method with a high sequence identity (99.60 and 99.49%) respectively, and full coverage (1.00) with their respective templates. Sequence similarity values were 0.63 for OmpV and 0.60 for LPS, indicating strong conservation. The Global Model Quality Estimation (GMQE) scores were 0.79 for OmpV and 0.88 for LPS, supporting the reliability of the predicted models for subsequent structural and functional analyses for further computational analyses. Following refinement, the quality of the OmpV and LPS protein models was assessed (Supplementary Figure S5; Supplementary Tables S4, S5). Based on comprehensive evaluation of GDT-HA, RMSD, MolProbity, clash score, poor rotamers, and Ramachandran favored regions (over 97% residues in core regions), model 3 was selected for OmpV, and model 5 for LPS, as they demonstrated the most balanced and optimal structural characteristics, and confirm the high quality and reliability of these models.

**Figure 2:**
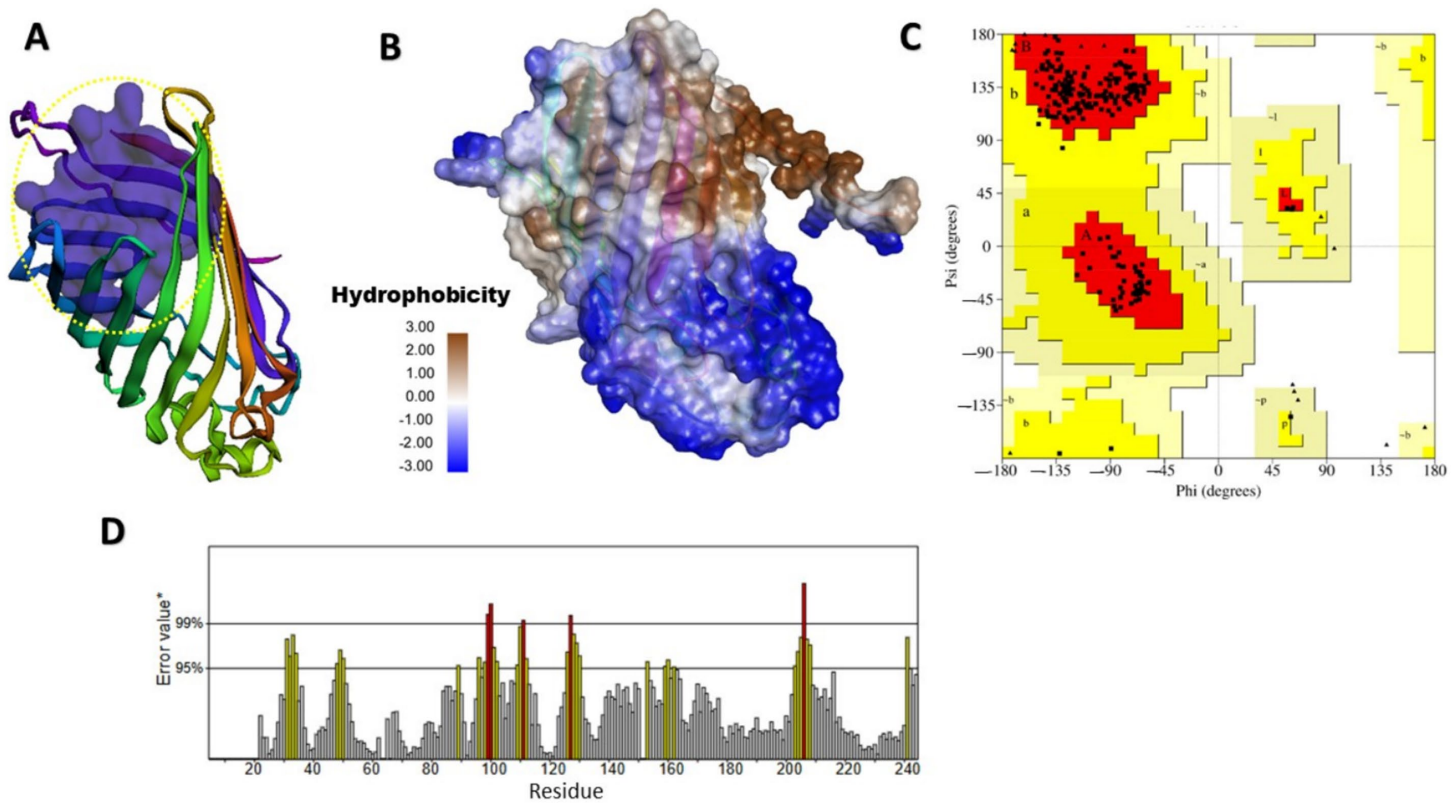
Modeled and structural quality assessment of MipA/OmpV family protein. **(A)** Identification of a rigid, exclusively apolar druggable pocket (Score: 0.998) in OmpV and hydrophobicity (score: 12.839) for lipid-Based ligand binding (Yellow dashed circular); **(B)** Hydrophobicity; **(C)** Ramachandran plot analysis, created using SAVES v6.1 web tool; **(D)** OmpV protein ERRAT evaluation obtained from the SAVES v6.1 web tool.

**Figure 3:**
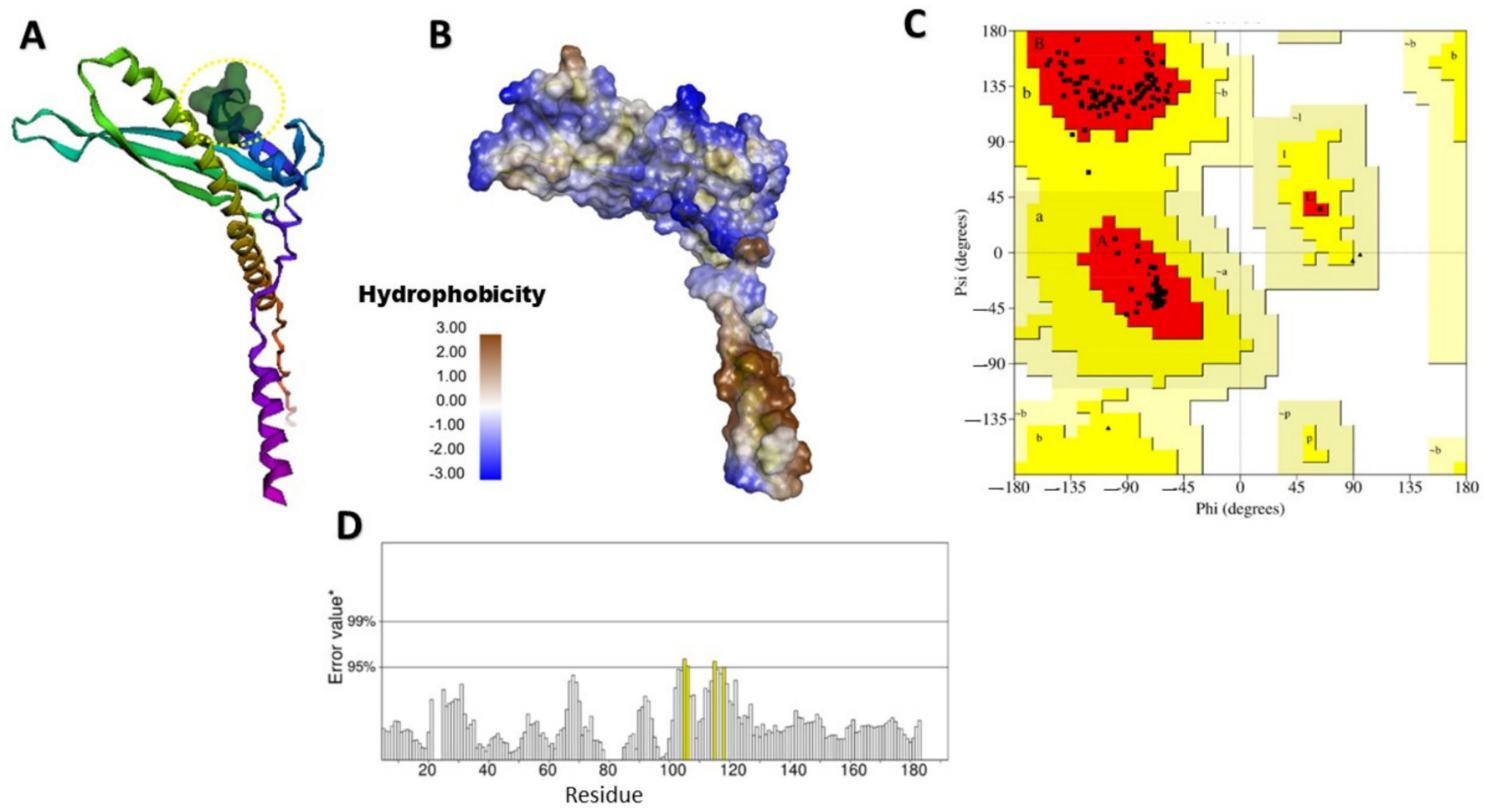
Modeled and structural quality assessment of LPS-assembly lipoprotein LptE. **(A)** Identification of a rigid, exclusively apolar druggable pocket (Score: 0.758) in LPS and hydrophobicity (score: −20.00) for lipid-Based ligand binding (Yellow dashed circular); **(B)** Hydrophobicity; **(C)** Ramachandran plot analysis, created using SAVES v6.1 web tool; **(D)** LPS protein ERRAT evaluation obtained from the SAVES v6.1 web tool.

Finding specific binding sites on target proteins is crucial for designing small-molecule drugs. Figures 2, 3 illustrate the predicted and structural quality assessment for OmpV and LPS proteins, respectively, identified using FPocketWeb 1.0.1. Analysis of these pockets (Supplementary Tables S7,S8) revealed that OmpV has a druggable pocket (0.998), while LPS possesses (0.758) respectively. Further validation with PROCHECK (Supplementary Tables S6,S7) confirmed the excellent stereochemical quality of both refined models, with over 97% of residues in core Ramachandran regions and no bad contacts, confirming their suitability for molecular docking studies. FPOCKETWEB analysis showed that both Ompv and LPS proteins have druggable pockets, scoring 0.998 and 0.758, respectively.

**Figure 4:**
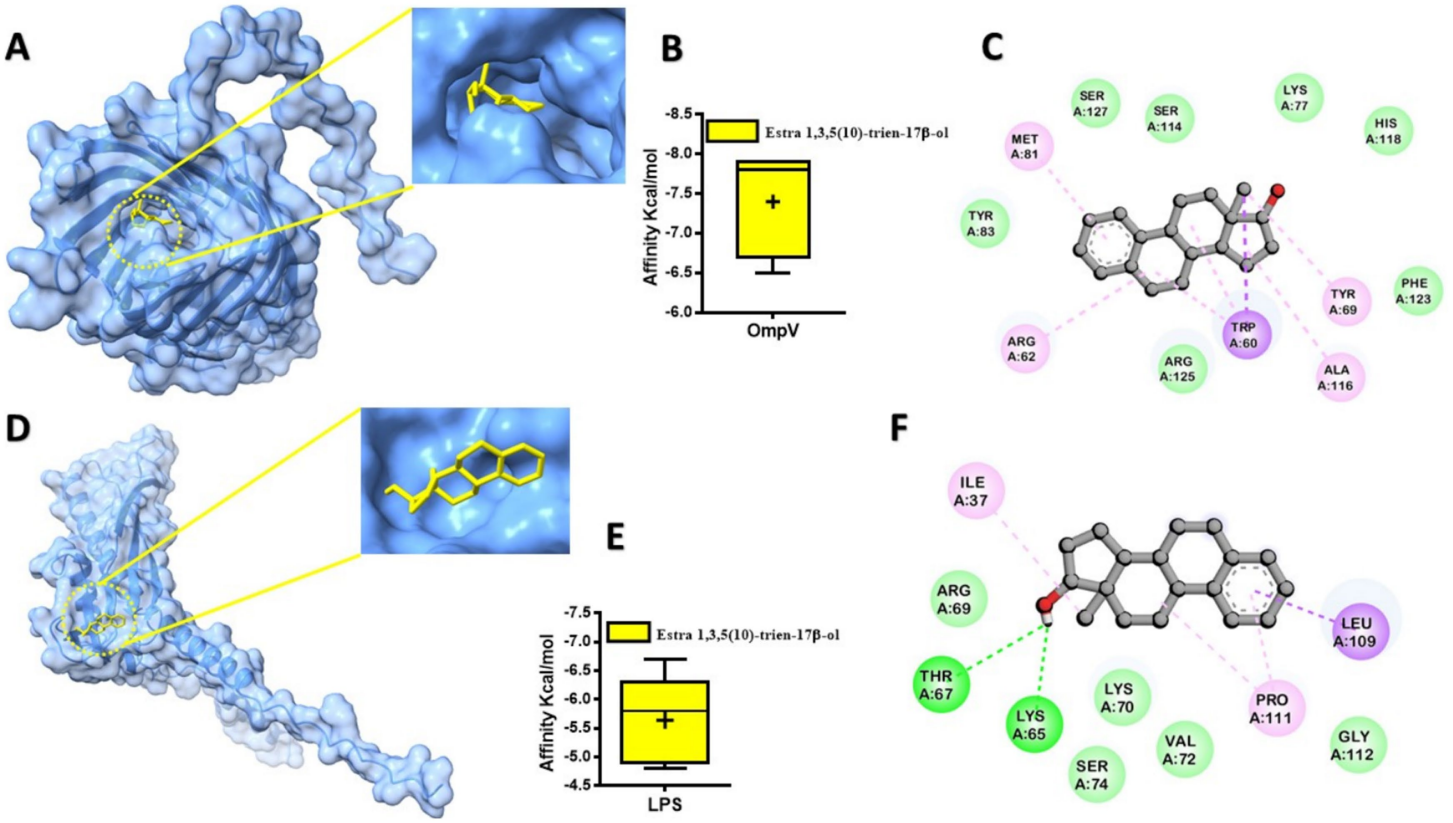
Predicted protein-ligand interaction. The docked compounds are shown in a stick model, colored yellow. **(A)** *A modeled* in OmpV docked with Estra-1,3,5(10)-trien-17β-ol; **(B)** *box plot represented binding affinity scores from computational predictions of* Estra-1,3,5(10)-trien-17β-ol*’s interaction with OmpV protein*.; **(C)** *2D* interaction of ligand with key residues; **(D)** human LPS docked with Estra-1,3,5(10)-trien-17β-ol; **(E)** *box plot represented binding affinity scores from computational predictions of* Estra-1,3,5(10)-trien-17β-ol*’s interaction with LPS protein*; **(F)** *2D* interaction of ligand with key residues. With the box plot showing a relatively tight distribution of predicted poses around this value.

Molecular docking simulations were performed to investigate the binding interactions between Solenostemma argel-derived compounds and the OmpV and LPS proteins, with binding affinities summarized in Table 7. Estra-1,3,5(10)-trien-17β-ol (Compound No. 3) emerged as the most promising candidate, exhibiting the highest binding affinities of −7.9 kcal/mol (estimated Ki = 1.65 μM) for OmpV and −6.7 kcal/mol (estimated Ki = 12.0 μM) for LPS. The lower Ki value for OmpV suggests a stronger potential inhibition of this outer membrane protein. Other compounds also showed notable binding, with Phenol, 2,4-bis(1,1-dimethylethyl)- and Phytol both achieving −6.6 kcal/mol against OmpV, and 4H-Pyran-4-one, 2,3-dihydro-3,5-dihydroxy- showing −5.1 kcal/mol against LPS. Figure 4 visually represents these interactions, showing the docked conformation of Estra-1,3,5(10)-trien-17β-ol within the binding pockets of OmpV (Figure 4A) and LPS (Figure 4D). The corresponding box plots (Figure 4B for OmpV and Figure 4E for LPS) illustrate a relatively tight distribution of predicted binding affinity scores, indicating consistent and stable binding poses. Furthermore, 2D interaction maps (Figures 4C,F) detail the key amino acid residues involved in the interactions of Estra-1,3,5(10)-trien-17β-ol with OmpV and LPS, respectively. LptE, on the other hand, is essential for the proper assembly of LPS in the outer membrane, which is vital for maintaining membrane integrity and facilitating LPS transport (19), making them attractive antibacterial targets. Estra-1,3,5(10)-trien-17β-ol demonstrates binding affinity to both OmpV and LPS suggests could has a potential mechanism for disrupting the outer membrane defense systems in Salmonella species. Computational docking analyses further elucidated that these interactions are stabilized by the formation of hydrogen bonds and hydrophobic region with amino acid residues within the binding sites. In particular, it has been reported that compounds such as estradiol and estrogen have antimicrobial effects, including membrane-targeting activity in *P. aeruginosa* biofilm structure (51), possibly interfere with bilayers or interfering with efflux and transport proteins. The double dubious capacity seen in this study highlights the ability of the connection for multi-scarcity bans, which can help reduce bacterial resistance development. However, further *in vitro* and *in vivo* studies are needed to confirm its efficacy.

**Table 7:** Molecular docking scores (ΔG) and estimated inhibition constants (Ki) of *S. argel*-derived compounds.

Compound name	OmpV*		LPS**	
	kcal/mol	μM	kcal/mol	μM
1,3-Dioxane	−3.5	2709.7	−3.3	3798.5
Phenol, 2,4-bis(1,1-dimethylethyl)-	−6.6	14.4	−5.4	109.5
Estra-1,3,5(10)-trien-17β-ol	−7.9	1.6	−6.7	12.2
4H-Pyran-4-one, 2,3-dihydro-3,5-dihydroxy-	−4.9	254.7	−5.1	181.7
Hexadecanoic acid, methyl ester	−5	215.2	−4.2	830.8
Phytol	−6.6	14.4	−5.5	92.5
Oleic Acid	−5.6	78.1	−4.4	592.7
2-Methoxy-4-vinylphenol	−5.5	92.5	−4.5	500.6
2-Furancarboxaldehyde, 5-(hydroxymethyl)-	−4.6	422. 8	−4.1	983.7
2-[2-[2-[2-(2-Acetyloxyethoxy)ethoxy]ethoxy]ethanol	−4.5	500.6	−3.9	1378.9
2-Acetylamino-3-hydroxy-propionic acid	−4.8	301.6	−4.5	500.6
(E)-Stilbene	−6.8	10.3	−5.2	153.5
12-Octadecadienoyl chloride, (Z, Z)-	−6	39.7	−4.9	254.7
2-Propanol, 1-(1-methylethoxy)-	−4.1	983.7	−3.8	1632.7
2-Methyl-Z, Z, Z-3,13-octadecadienol	−5.8	55.7	−4	1164.7
n-Hexadecanoic acid	−5.7	65.9	−4	1164.7
9,12,15-Octadecatrienoic acid	−6.1	33.6	−4.7	357.1
Pentanoic acid, 3-methyl-, methyl ester	−4.5	500.6	−4.3	701.7
6-Methyl-2,3-dihydro-pyran-2,4-dione	−4.8	301.6	−5.1	181.7

MipA/OmpV family protein; *OmpV: Outer membrane protein V. **LPS-assembly lipoprotein LptE. Ki values were calculated from the docking scores (ΔG) using the equation Ki = exp.(ΔG/RT), where R = 1.987 × 10^−3^ kcal·mol^−1^·K^−1^ and T = 298.15 K. Ki values are reported in micromolar (μM).

Studies on *S. argel* have shown antimicrobial, antioxidant, anti-inflammatory, and anticancer activities. Here is not much direct proof that Estra-1,3,5(10)-trien-17β-ol works against Salmonella or interacts with OmpV/LPS, but its steroidal structure, which is similar to estrogen, suggests that it could have a wide range of biological effects.

### 3.5 Limitations of the study

While this study provides a quantitative baseline for the anti-*Salmonella* activity of *S. argel* methanolic leaf extract, several limitations should be acknowledged. First, the work was conducted exclusively *in vitro*, relying on disc diffusion and microdilution assays. Consequently, the results do not demonstrate whether the extract retains activity under more complex conditions such as real food matrices (for example, chicken meat during refrigeration) or *in vivo* environments such as the animal or human gastrointestinal tract. Stability of the active compounds, their potential sensory impacts, and interactions with food components were not assessed, and these factors are critical for practical application in food safety. Second, the phytochemical characterization was restricted to GC–MS profiling, which primarily captures volatile and semi-volatile constituents. Non-volatile but potentially bioactive metabolites such as polyphenols, alkaloids, or glycosides may have been overlooked, limiting the comprehensiveness of the chemical analysis. Third, all assays were performed with crude methanolic extract; the reported MIC and MBC values therefore reflect the combined action of multiple constituents and do not allow identification of specific active principles or potential synergistic effects. Fourth, the microbiological panel was relatively limited, comprising only three *Salmonella enterica* isolates, which restricts generalization across diverse serovars and clinically relevant resistant strains. Finally, the *in-silico* component relied on homology modelling and docking predictions, which are valuable for hypothesis generation but cannot confirm biochemical binding or functional inhibition without experimental validation.

It is also important to note that some compounds detected in the GC–MS analysis, such as 1,3-dioxane (14.07%), are highly likely to represent contaminants introduced from organic solvents or plasticware rather than authentic plant-derived metabolites. Similarly, signals corresponding to compounds such as 2-[2-[2-(2-acetyloxyethoxy) ethoxy] ethoxy] ethanol are suspected to have originated from laboratory consumables or leaching during storage in plastic containers. Because we did not carry out complementary confirmatory analyses (for example, LC–MS/MS with authentic standards or alternative extraction and storage conditions), we cannot fully exclude the contribution of such artifacts. Recognizing this limitation is critical to avoid overinterpretation of the GC–MS data. We therefore recommend that future investigations incorporate rigorous contamination controls, use glassware and solvent blanks, and apply orthogonal analytical techniques to distinguish genuine phytochemicals from external impurities. Addressing this point will ensure greater accuracy in the phytochemical profiling of *S. argel* and strengthen the reliability of subsequent bioactivity correlations.

### 3.6 Future perspectives

To translate these preliminary findings into validated antimicrobial leads and practical interventions, a staged, mechanistic program of work is recommended. The immediate priority should be bioactivity-guided fractionation of the methanolic extract (liquid–liquid partitioning followed by chromatographic separation) to isolate and structurally characterize the compounds responsible for antibacterial activity; identities must be confirmed by LC–MS/MS and NMR. Concurrently, focused mechanistic assays are needed to test the membrane-targeting hypothesis suggested by GC–MS and docking: these should include membrane-integrity assays (e.g., propidium iodide uptake, membrane potential probes), LPS-interaction or displacement assays, and efflux-pump activity measurements. Given the public-health importance of antibiotic resistance, systematically testing synergy with frontline antibiotics (FICI determinations for ciprofloxacin, ceftriaxone, ampicillin, and others) will establish whether *S. argel* fractions can potentiate existing drugs or mitigate resistance. Target validation should move beyond docking to biochemical and biophysical binding assays (for example, SPR or ITC) using recombinant outer-membrane proteins prioritized by *in silico* screens. Once active fractions or pure compounds are identified, *in vivo* evaluation in an appropriate *Salmonella* infection model (avian or murine, depending on the intended application) is essential to characterize efficacy, dosing, and safety; standard toxicology panels and histopathology must accompany these studies. Finally, because solvent selection and extraction methodology strongly influence phytochemical yield and bioactivity, parallel optimization of extraction (testing solvents such as ethyl acetate, diethyl ether, and supercritical CO₂) should be performed to maximize recovery of potent constituents and to develop reproducible preparations suitable for downstream formulation and scale-up.

## 4 Conclusion

In conclusion, this study demonstrates that the methanolic leaf extract of *Solenostemma argel* exhibits moderate *in vitro* activity against the tested Salmonella strains. The GC–MS profile, rich in phenolic constituents and fatty-acid derivatives, lends phytochemical support to the plant’s ethnobotanical use and suggests mechanisms consistent with antimicrobial and antioxidant effects. Because the traditional use of *S. argel* for abdominal complaints may not reflect direct antibacterial action alone, our findings underscore the need to evaluate other physiological and host-directed effects and to test efficacy in appropriate *in vivo* models. Importantly, antibacterial principals may be polar and therefore poorly recovered by the present methanolic protocol; consequently, systematic studies using alternative solvents and extraction techniques are warranted. The detection of possible analytical artifacts also highlights the necessity of rigorous sample handling and orthogonal compound confirmation (LC–MS/MS, NMR). Ultimately, bioactivity-guided isolation and structural characterization of individual compounds, followed by mechanistic and *in vivo* validation, will be essential to determine the true therapeutic potential of *S. argel* and to explore its promise as a source of novel anti-*Salmonella* agents for food-safety applications, particularly in poultry production.

## Data Availability

The original contributions presented in the study are included in the article/Supplementary material, further inquiries can be directed to the corresponding authors.

## Footnotes

^1^ https://tox.charite.de/protox3/

^2^ https://www.uniprot.org/

^3^ https://swissmodel.expasy.org/

^4^ https://saves.mbi.ucla.edu/

^5^ https://cadd.labshare.cn/cb-dock2/index.php
