# Supplementary material for: Antibacterial properties of Solenostemma argel (Del.) Hayne against Salmonella strains from chicken meat: integrated GC–MS phytochemical profiling and molecular docking analysis
Source: Front Nutr. 2025 Oct 29;12:1694017. doi: 10.3389/fnut.2025.1694017 (PMC12614465; doi:10.3389/fnut.2025.1694017)
Supplement: Supplementary file 1 [file Data_Sheet_1.docx]

Supplementary Material

# Supplementary Figures and Tables

**1.1 Supplementary Figures**


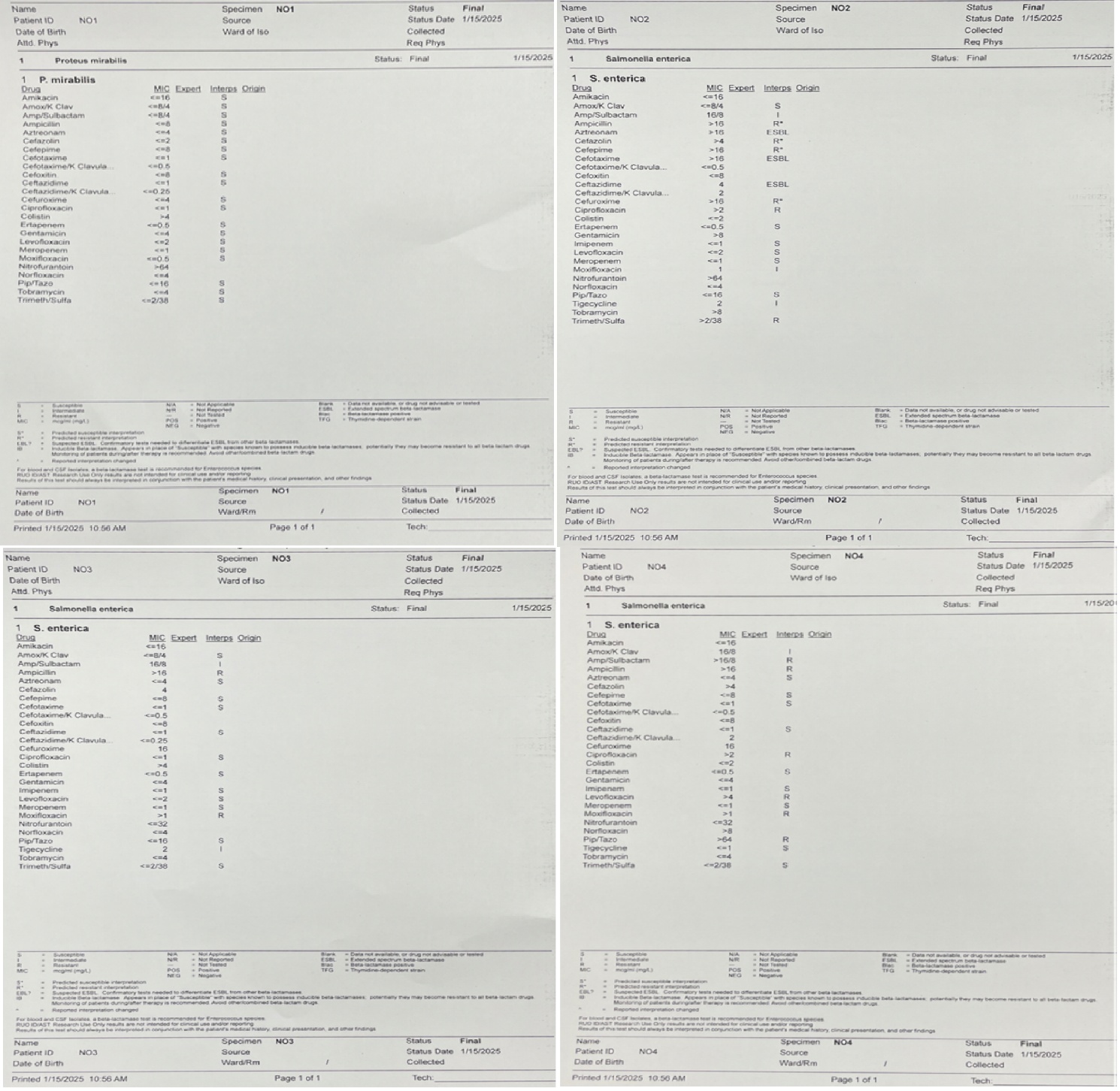


## Figure S1. Automated identification and antimicrobial susceptibility profiling of presumptive *Salmonella* Isolates.


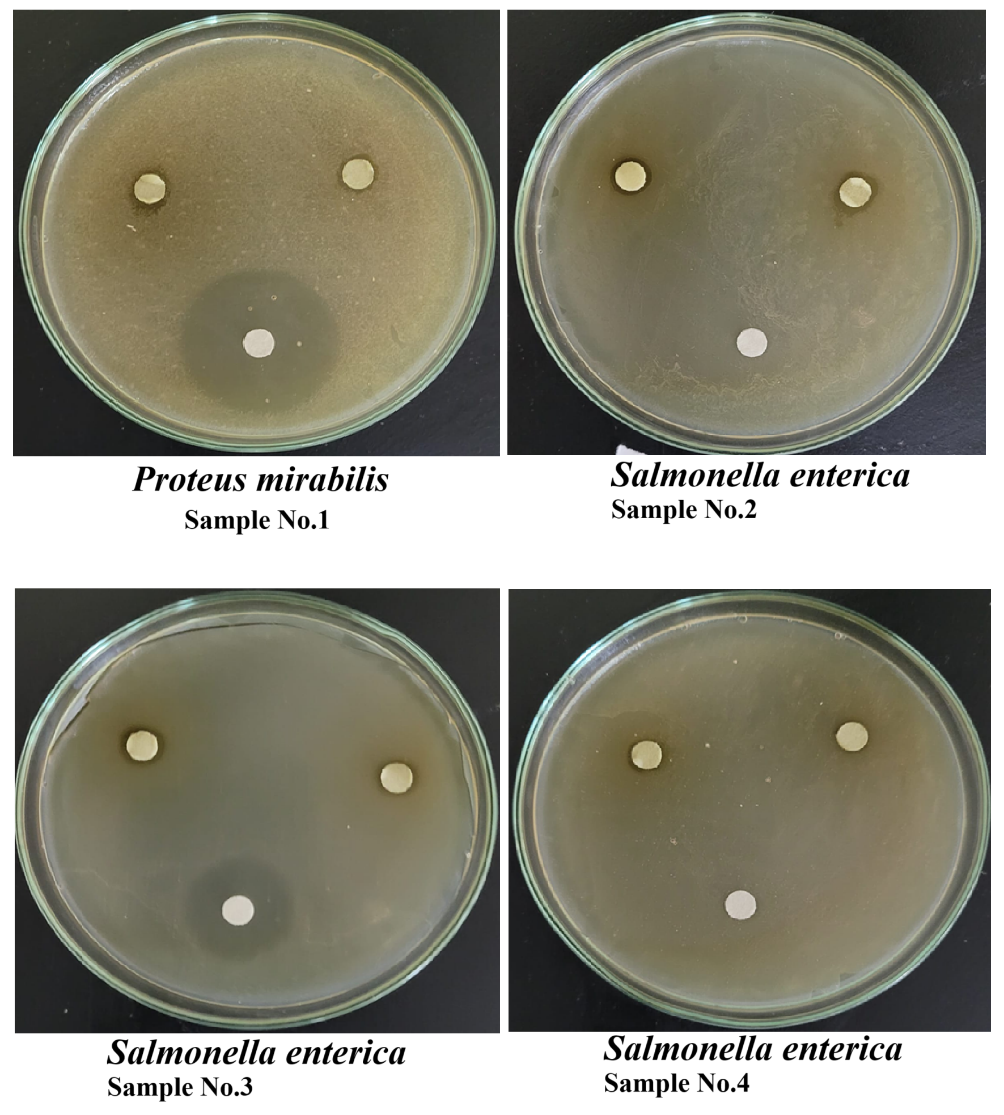


**Figure S2.** Representative photo showing moderate zones of inhibitions of the tested bacteria around the discs saturated with *S. argel* methanolic extracts


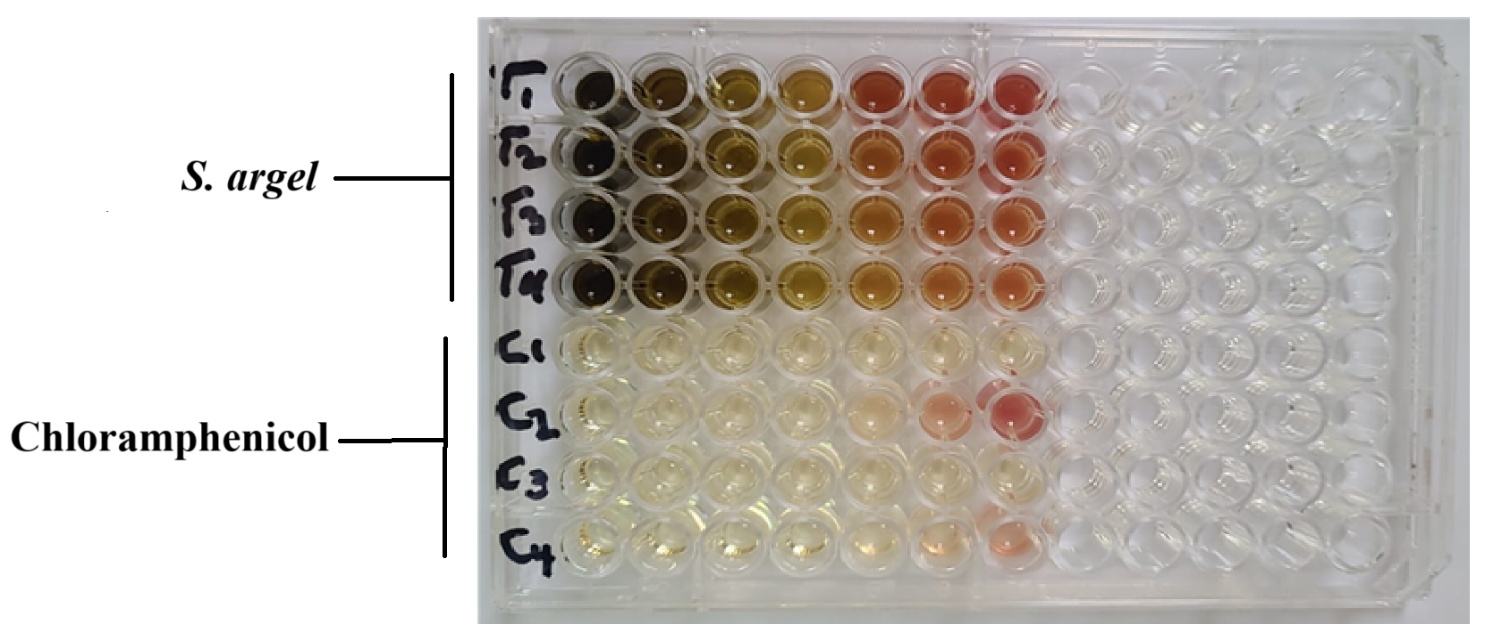


**Figure S3.** Representative photo showing the MIC test.


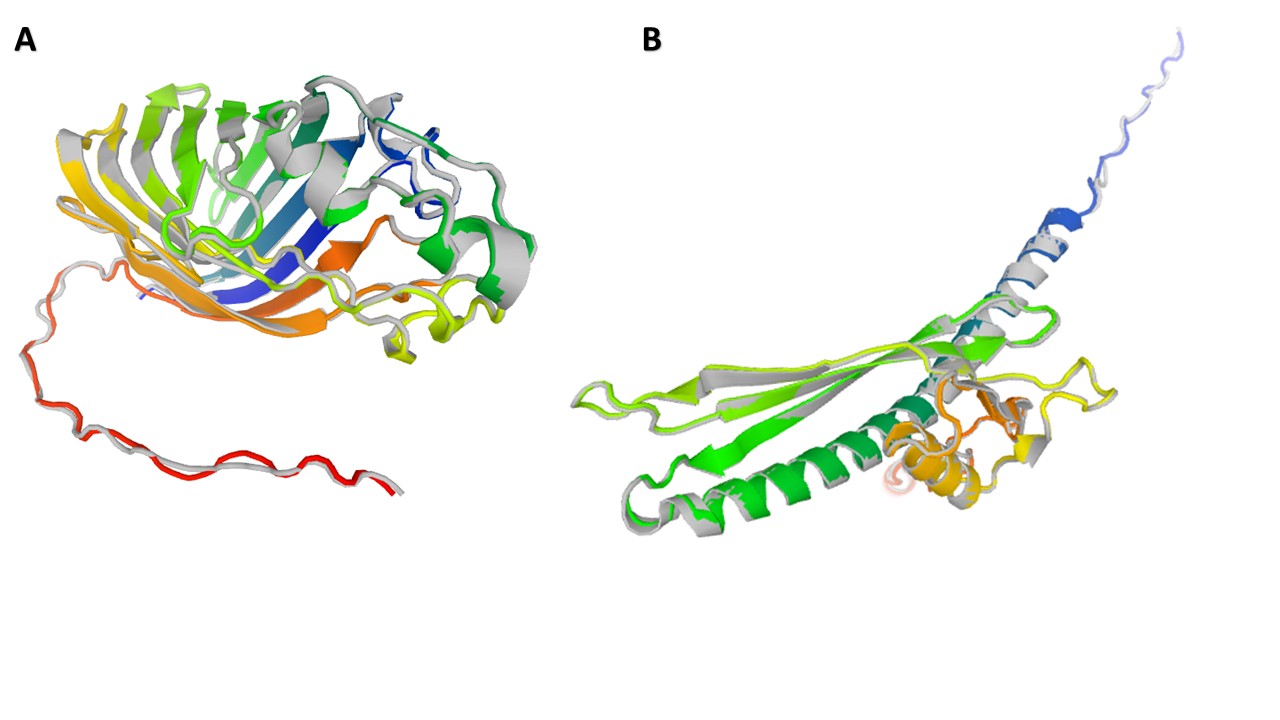


**Figure S5: Validation of Refined *Salmonella* Protein Structure.** (A): MipA/OmpV family protein; (B) LPS-assembly lipoprotein LptE.

- 1. **Supplementary Tables**

Table S1**:** Simplified Molecular Input Line Entry System (SMILES) of *Rhazya stricta*-derived compounds.

| **No** | **Compound** | **SMILES** |
| --- | --- | --- |
| 1 | 1,3‑Dioxane | C1COCOC1 |
| 2 | Phenol, 2,4‑bis(1,1‑dimethylethyl)- | CC(C)(C)C1=CC(=CC=C1C(C)(C)C)O |
| 3 | Estra‑1,3,5(10)-trien-17β-ol | CC12CCC3C(C1CCC2O)CCC4=CC=CC=C34 |
| 4 | 4H‑Pyran‑4‑one, 2,3‑dihydro-3,5-dihydroxy- | CC1=C(O)C(=O)C(O)CO1 |
| 5 | Hexadecanoic acid, methyl ester | CCCCCCCCCCCCCCCC(=O)OC |
| 6 | Phytol | CC(C)=CCCC(C)CC/C=C(/C)/CC/C=C/C/C(O)C |
| 7 | Oleic Acid | CCCCCCCC=CCCCCCCCC(=O)O |
| 8 | 2‑Methoxy-4-vinylphenol | COC1=CC=C(C=C1)C=C |
| 9 | 2‑Furancarboxaldehyde, 5-(hydroxymethyl)- | O=CC1=COC=C1CO |
| 10 | 2‑[2-[2-[2-(2-Acetyloxyethoxy)ethoxy]ethoxy]ethanol | CC(=O)OCCOCCOCCO |
| 11 | 2‑Acetylamino-3-hydroxy-propionic acid | CC(=O)NC(CO)C(=O)O |
| 12 | (E)-Stilbene | C1=CC=C(C=C1)/C=C/C2=CC=CC=C2 |
| 13 | 12‑Octadecadienoyl chloride, (Z,Z)- | O=C(Cl)CCCCCCCC=CCC=CCCCCC |
| 14 | 2‑Propanol, 1-(1-methylethoxy)- | CC(C)OCC(C)O |
| 15 | 2‑Methyl-Z,Z,Z-3,13-octadecadienol | CCCC/C=C/CCCCCCCC/C=C\C(C)CO |
| 16 | n‑Hexadecanoic acid | CCCCCCCCCCCCCCCC(=O)O |
| 17 | 9,12,15‑Octadecatrienoic acid | CC/C=C/CC/C=C/CC/C=C/CCCC(=O)O |
| 18 | Pentanoic acid, 3-methyl-, methyl ester | CCC(C)CC(=O)OC |
| 19 | 6‑Methyl-2,3-dihydro-pyran-2,4-dione | CC1=C(O)C(=O)C(O)CO1 |

| **Compound No.** | **Hepatotoxicity** | | **Carcinogenicity** | | **Immunotoxicity** | | **Mutagenicity** | | **Cytotoxicity** | |
| --- | --- | --- | --- | --- | --- | --- | --- | --- | --- | --- |
|  | **Pred** | **Prob** | **Pred** | **Prob** | **Pred** | **Prob** | **Pred** | **Prob** | **Pred** | **Prob** |
| 1 | Inactive | 0.86 | Active | 0.74 | Inactive | 0.99 | Active | 0. 37 | Inactive | 0.82 |
| 2 | Inactive | 0.78 | Inactive | 0.52 | Inactive | 0.93 | Inactive | 0.99 | Inactive | 0.91 |
| 3 | Inactive | 0.72 | Active | 0.85 | Active | 0.95 | Inactive | 0.95 | Inactive | 0.86 |
| 4 | Inactive | 0.80 | Inactive | 0.70 | Inactive | 0.97 | Active | 0.78 | Inactive | 0.77 |
| 5 | Inactive | 0.85 | Inactive | 0.55 | Inactive | 0.99 | Inactive | 0.98 | Inactive | 0.73 |
| 6 | Inactive | 0.78 | Inactive | 0.70 | Inactive | 0.96 | Inactive | 0.95 | Inactive | 0.86 |
| 7 | Inactive | 0.55 | Inactive | 0.64 | Inactive | 0.99 | Inactive | 1.00 | Inactive | 0.71 |
| 8 | Inactive | 0.72 | Active | 0.50 | Inactive | 0.88 | Inactive | 0.96 | Inactive | 0.92 |
| 9 | Inactive | 0.77 | Active | 0.52 | Inactive | 0.99 | Inactive | 0.55 | Inactive | 0.82 |
| 10 | Inactive | 0.93 | Active | 0.52 | Inactive | 0.99 | Inactive | 0.66 | Inactive | 0.82 |
| 11 | Inactive | 0.90 | Inactive | 0.69 | Inactive | 0.99 | Inactive | 0.52 | Inactive | 0.67 |
| 12 | Inactive | 0.84 | Active | 0.54 | Inactive | 0.98 | Inactive | 0.98 | Inactive | 0.94 |
| 13 | Inactive | 0.74 | Inactive | 0.52 | Inactive | 0.88 | Active | 0.65 | Inactive | 0.70 |
| 14 | Inactive | 0.86 | Active | 0.61 | Inactive | 0.99 | Active | 0.54 | Inactive | 0.84 |
| 15 | Inactive | 0.80 | Inactive | 0.54 | Inactive | 0.97 | Inactive | 0.84 | Inactive | 0.84 |
| 16 | Inactive | 0.52 | Inactive | 0.63 | Inactive | 0.99 | Inactive | 1.00 | Inactive | 0.74 |
| 17 | Inactive | 0.54 | Inactive | 0.63 | Active | 0.99 | Inactive | 0.95 | Inactive | 0.71 |
| 18 | Inactive | 0.66 | Inactive | 0.52 | Inactive | 0.99 | Inactive | 0.90 | Inactive | 0.79 |
| 19 | Inactive | 0.80 | Inactive | 0.70 | Inactive | 0.97 | Active | 0.78 | Inactive | 0.77 |

Table S2. Organ toxicity prediction of *S. argel* compound from ProTox-II web server.

* Pred: Prediction; Prop: Probability.

**Table S3:** AlphaFold2-predicted structural characteristics of OmpV and LPS proteins.

| **Feature** | **OmpV*** | **LPS*** |
| --- | --- | --- |
| Template | A0A5I0D5G6.1.A | B5R7Z5.1.A |
| AlphaFold DB model | *Salmonella enterica* subsp enterica serovar Ouagadougou | *Salmonella gallinarum* (strain 287/91 / NCTC 13346) |
| Biounit oligo state | Monomer | Monomer |
| Method | AlphaFold v2 | AlphaFold v2 |
| Sequence similarity | 0.63 | 0.60 |
| Sequence identity | 99.60 | 99.49 |
| Coverage | 1.00 | 1.00 |
| Range | 1-248 | 1-196 |
| GMQE* | 0.79 | 0.88 |

*MipA/OmpV family protein; ** LPS-assembly lipoprotein LptE; *** Global model quality estimation.

**Table S4:** Evaluation of structural quality characteristics of OmpV and LPS proteins using MolProbity

| **Category** | **OmpV*** **value** | **LPS**** **value** |
| --- | --- | --- |
| MolProbity Score | 1.28 | 1.02 |
| Clash Score | 0.00 | 0.00 |
| Ramachandran Favored | 95.12% | 96.39 |
| Ramachandran Outliers | 0.41% | 1.033 |
| Ramachandran Outlier Residues | A9 LEU | A184 ALA, A187 PRO |
| Rotamer Outliers | 3.83% | 2.37% |
| Rotamer Outlier Residues | A16 SER, A9 LEU, A44 SER, A90 LYS, A15 THR, A7 LEU, A2 THR, A4 LEU | A120 THR, A14 LEU, A8 LEU, A186 THR |
| C-Beta Deviations | 2 | 1 |
| C-Beta Deviation Residues | A9 LEU, A73 ASP | A186 THR |
| Bad Bonds | 0/2043 | 0 / 1521 |
| Bad Bond Residue | - | - |
| Bad Angles | 10/2784 | 5 / 2070 |
| Twisted Prolines |  | 1/9 |
| Twisted Proline Residue |  | A186 THR-A187 PRO |
| Twisted Non-Prolines | 1/238 | 2/186 |
| Twisted Non-Proline Residues | A8 ALA-A9 LEU | A185 SER-A186 THR), (A195 SER-A196 ASN |

*MipA/OmpV family protein; ** LPS-assembly lipoprotein LptE.

**Table S5:** Refinement of structural quality characteristics of OmpV S protein.

| **Model** | **GDT-HA** | | **RMSD** | | **MolProbity** | | **Clash Score** | | **Poor Rotamers** | | **Rama Favored** | |  |
| --- | --- | --- | --- | --- | --- | --- | --- | --- | --- | --- | --- | --- | --- |
| Initial | | 1.0000 | | 0.000 | | 1.225 | | 0.3 | | 2.4 | | 95.1 | |
| MODEL 1 | | 0.9587 | | 0.421 | | 1.334 | | 6.1 | | 0.5 | | 99.2 | |
| MODEL 2 | | 0.9405 | | 0.475 | | 1.378 | | 6.9 | | 0.0 | | 99.6 | |
| MODEL 3 | | 0.9587 | | 0.434 | | 1.232 | | 4.6 | | 0.5 | | 99.2 | |
| MODEL 4 | | 0.9415 | | 0.495 | | 1.378 | | 6.9 | | 0.0 | | 99.6 | |
| MODEL 5 | | 0.9556 | | 0.432 | | 1.508 | | 9.7 | | 1.0 | | 99.6 | |

**Table S6:** Refinement of structural quality characteristics of LPS protein.

| **Model** | **GDT-HA** | | **RMSD** | | **MolProbity** | | **Clash Score** | | **Poor Rotamers** | | **Rama Favored** | |  |
| --- | --- | --- | --- | --- | --- | --- | --- | --- | --- | --- | --- | --- | --- |
| Initial | | 1.0000 | | 0.000 | | 1.222 | | 1.0 | | 1.8 | | 96.4 | |
| MODEL 1 | | 0.9592 | | 0.409 | | 1.169 | | 3.2 | | 1.2 | | 99.5 | |
| MODEL 2 | | 0.9796 | | 0.311 | | 1.173 | | 3.9 | | 0.0 | | 99.5 | |
| MODEL 3 | | 0.9681 | | 0.373 | | 1.282 | | 4.5 | | 1.2 | | 100.0 | |
| MODEL 4 | | 0.9821 | | 0.332 | | 1.229 | | 3.9 | | 1.2 | | 99.5 | |
| MODEL 5 | | 0.9694 | | 0.366 | | 1.079 | | 2.9 | | 0.6 | | 99.5 | |

**Table S7:** PROCHECK validation of refined protein models.

| **Category** | **OmpV*** **value** | **LPS**** **value** |
| --- | --- | --- |
| Total Residues | 248 | 196 |
| Ramachandran Plot | 97.7% core, 2.3% allowed, 0.0% generously allowed, 0.0% disallowed | 98.3% core, 1.7% allowed, 0.0% generously allowed, 0.0% disallowed |
| Labelled Residues | 5 out of 246 | 5 out of 196 |
| Chi1-chi2 Plots | 0 labelled residues (out of 145) | 0 labelled residues (out of 107) |
| Side-chain Params | 5 better, 0 inside, 0 worse | 5 better, 0 inside, 0 worse |
| Max. Deviation | 4.0 Å | 2.3 Å |
| Bad Contacts | 0 | 0 |
| Bond len/angle | 6.1 | 4.4 |
| Morris et al Class | 1, 1, 2 | 1, 1, 1 |
| G-factors | Dihedrals: 0.24, Covalent: -0.19, Overall: 0.08 | Dihedrals: 0.53, Covalent: -0.09, Overall: 0.29 |
| Planar Groups | 100.0% within limits, 0.0% highlighted | 100.0% within limits, 0.0% highlighted |

*MipA/OmpV family protein; ** LPS-assembly lipoprotein LptE.

**Table S8:** FPocketWeb Analysis of OmpV and LPS proteins Binding Pocket Properties.

| **Category** | **OmpV*** **value** | **LPS**** **value** |
| --- | --- | --- |
| Score | 1.455 | 0.742 |
| Druggability Score | 0.998 | 0.758 |
| Number of Alpha Spheres | 134 | 24 |
| Total SASA******* | 66.371 | 12.225 |
| Polar SASA | 0.000 | 0.000 |
| Apolar SASA | 66.371 | 12.225 |
| Volume | 1770.962 | 298.830 |
| Mean local hydrophobic density | 54.134 | 21.417 |
| Mean alpha sphere radius | 4.014 | 3.805 |
| Mean alpha sphere solvent access | 0.572 | 0.590 |
| Apolar alpha sphere proportion | 1.000 | 1.000 |
| Hydrophobicity score | 12.839 | -20.00 |
| Volume score | 4.290 | 3.571 |
| Polarity score | 26 | 5 |
| Charge score | 3 | 0 |
| Proportion of polar atoms | 0.000 | 0.000 |
| Alpha sphere density | 9.450 | 3.730 |
| Cent. of mass - Alpha Sphere max dist | 26.031 | 8.746 |
| Flexibility | 0.000 | 0.000 |

*MipA/OmpV family protein; ** LPS-assembly lipoprotein LptE; *** Solvent accessible surface area.
